# Current implementation and perception of palliative interventional radiology procedures for patients with refractory cancer pain among interventional radiologists in Japan: a nationwide survey

**DOI:** 10.1007/s11604-026-01961-3

**Published:** 2026-03-03

**Authors:** Miyuki Sone, Yoshihisa Matsumoto, Yuko Uehara, Toshifumi Kosugi, Naoki Nakamura, Akio Mizushima, Mitsunori Miyashita, Tatsuya Morita, Takuhiro Yamaguchi, Eriko Satomi

**Affiliations:** 1 Department of Diagnostic Radiology/Interventional Radiology Center, National Cancer Center Hospital, 5-1-1, Tsukiji, Chuo-ku, Tokyo, 1040045 Japan; 2 Department of Palliative Therapy, Cancer Institute Hospital of JFCR, Tokyo, Japan; 3 Department of Palliative Medicine, Juntendo University Graduate School of Medicine, Tokyo, Japan; 4 Department of Palliative Care, Saga-Ken Medical Centre Koseikan, Saga, Japan; 5 Department of Radiology, St. Marianna University School of Medicine, Kawasaki, Japan; 6 Department of Palliative Nursing, Health Sciences, Tohoku University Graduate School of Medicine, Sendai, Japan; 7 Division of Supportive and Palliative Care, Seirei Mikatahara General Hospital, Hamamatsu, Japan; 8 Division of Biostatistics, Tohoku University School of Medicine, Sendai, Japan; 9 Department of Palliative Medicine, National Cancer Center Hospital, Tokyo, Japan

**Keywords:** Refractory cancer pain, Interventional radiology, Nationwide survey, Osteoplasty, Nerve block, Transcatheter arterial embolization

## Abstract

**Purpose:**

Palliative interventional radiology (IR) procedures are effective for managing refractory cancer pain, but their real-world utilization—including procedural volume, physicians’ perceptions, and barriers to implementation—remains unclear. This study aimed to assess the current implementation of these procedures in Japan and to explore IR physicians’ perceptions and barriers.

**Materials and methods:**

A nationwide cross-sectional survey was conducted among 1,087 board-certified IR physicians in Japan from February to March 2020. A self-administered questionnaire assessed current practice for three procedures (celiac plexus/splanchnic nerve block [nerve block], percutaneous vertebroplasty/osteoplasty [bone augmentation], and transcatheter arterial embolization [TAE]), perceived effectiveness, willingness to perform in the future, and demographics. Barriers to implementation, which were predefined as questionnaire items, were evaluated using univariable and multivariable logistic regression analyses.

**Results:**

A total of 554 valid responses were obtained (response rate: 51.1%). Although all respondents were board-certified IR physicians, 52.5% reported diagnostic imaging as their primary practice, and 35.0% were dedicated to IR. Implementation rates were 18.1% for nerve block, 22.6% for bone augmentation, and 51.4% for TAE. Lack of adequate training was the most consistent barrier across all procedures, along with delayed referrals, lack of confidence, limited case volume, time constraints, institutional resource limitations, and insufficient evidence. Perceived effectiveness was high (81.2% for nerve block, 83.1% for bone augmentation, and 62.1% for TAE), whereas willingness to perform was lower (50.2%, 48.4%, and 66.0%).

**Conclusion:**

Implementation of palliative IR procedures in Japan remains limited, despite the high perceived effectiveness among IR physicians. Lack of adequate training was the most consistent barrier across all procedures, suggesting that greater availability of training opportunities may help narrow the gap between perceived benefit and actual practice. These findings highlight a clinically important mismatch: procedures viewed as effective for refractory cancer pain are not reaching patients who might benefit from them.

## Introduction

Pain remains a significant issue for cancer patients, occurring in 40 − 53% across all disease stages [1–3] and 66.4% in advanced disease [3]. When adequate pain relief cannot be achieved despite appropriate pharmacotherapy, the condition is referred to as refractory cancer pain, affecting an estimated 10–20% of patients [4, 5]. For these patients, nonpharmacological treatments play an important complementary role in pain management and have been shown to provide effective symptom relief [6–10].

Despite their potential benefits, previous nationwide surveys have indicated that nonpharmacological interventions for cancer pain are underutilized and do not sufficiently meet clinical demand [11, 12]. Reported contributing factors include low procedural volumes, limited training opportunities, and insufficient institutional support [11, 12]. Among nonpharmacological approaches, interventional radiology (IR) offers image-guided, target-oriented treatments that directly address pain generators, making it a promising option for refractory cancer pain. Representative palliative IR procedures include nerve blocks, bone augmentation procedures, transcatheter arterial embolization, and thermal ablation, each of which can be tailored to the underlying pain mechanism. The demand for IR continues to grow [13]; however, access to palliative IR procedures remains inconsistent. Palliative care physicians and oncologists, who often serve as primary decision-makers for pain management, may have limited awareness of IR options or of established referral pathways, which can hinder timely consultation [11]. In addition, the implementation of palliative IR procedures varies widely across institutions, reflecting differences in available expertise, infrastructure, and organizational support [11, 12, 14]. While these system-level barriers have been partially described, less is known about the extent to which IR physicians currently provide palliative IR procedures or about their willingness to do so in routine practice. Therefore, this study aimed to assess the current implementation of IR for refractory cancer pain in Japan and to explore IR physicians’ perceptions and perceived barriers to providing these procedures.

## Materials and methods

### Study design

This quantitative, cross-sectional survey on palliative IR procedures in Japan was conducted as part of the “Research on the Construction of Systematic Pain Relief Methods in the Final Stage of Cancer Patients’ Medical Care [11].” The protocol was approved by the Institutional Review Board of the National Cancer Center, Japan (6000-021). In accordance with national policy, additional approval from individual institutions was not required. The overall project included pain specialists, interventional radiologists, home hospice physicians, and clinical oncologists. Results of nerve block implementation by pain specialists have been published separately [11]. The present study specifically examined three IR procedures and factors influencing their implementation by IR physicians, which were not addressed in the previous report.

### Study population and survey methods

From February to March 2020, a questionnaire, cover letter, and return envelope were mailed to all 1,087 board-certified IR physicians in Japan, with one reminder sent to non-respondents. Participation was voluntary, and questionnaire completion implied consent. Responses were anonymous and confidential, and no incentives were offered.

### Questionnaire items (supplementary material file of the questionnaire)

The authors developed, reviewed, and pre-tested the questionnaire items. Content clarity and relevance were reviewed by three board-certified interventional radiologists. A pre-test was conducted to evaluate comprehensibility and response burden, including completion time, and minor revisions were made accordingly. The questionnaire included 19 questions on four domains: (i) the current implementation of three IR procedures for cancer pain (celiac plexus/splanchnic nerve block [nerve block], percutaneous vertebroplasty/osteoplasty [bone augmentation], and transcatheter arterial embolization [TAE]), ); (ii) current acceptance and barriers to performing the three IR procedures; (iii) future willingness to performing the three IR procedures; (iv) demographics of the participants.

### Statistical analysis

Descriptive statistics were used to summarize responses. Willingness was dichotomized into *willing* (“Yes, we would like to continue performing it.” and “Yes, we would like to start performing it.” and *unwilling* (“Prefer not to perform it if possible.” or “No, we do not plan to perform it.”). Likert scale items were dichotomized into *agree* (“strongly agree,” “agree,” “somewhat agree”) and *neutral/disagree* (all others). Associations with procedure implementation were examined using chi-square or Fisher’s exact test. Independent predictors were evaluated using logistic regression analyses. Variables showing *p* < 0.05 in univariable analyses were entered into the multivariable models, together with sex and age group, which were included a priori. Odds ratios (ORs) with 95% confidence intervals (CIs) were calculated, and *p* < 0.05 was considered statistically significant. Cases with missing data were excluded from the corresponding analyses. No formal sample size or power calculation was performed because this study was designed as an exploratory nationwide survey. All analyses were performed using SPSS Statistics, version 29 (IBM Corp., Armonk, NY, USA).

## Results

Response rate

Of the 1,087 physicians invited, 572 responded; 18 declined participation, resulting in 554 valid responses (response rate: 51.1%).

Demographics of Respondents.

The respondents’ characteristics are listed in Table 1. Respondents were distributed across a wide age range, with a mean age of 48.2 ± 9.5 years. The median number of cancer patients with pain treated with IR was 3 (inter-quartile range: 0–10) annually, indicating that most respondents had limited clinical experience with palliative IR procedures. Although all respondents were board-certified interventional radiologists, 52.5% reported diagnostic radiology as their primary practice area, while 35.0% primarily practiced interventional radiology.

**Table 1:** Respondent demographics and practice characteristics (*N* = 554)

Characteristic	
*Gender*	
Male	510 (92.1%)
Female	42 (7.6%)
No answer	2 (0.4%)
*Age, years*	
Mean ± SD	48.2 ± 9.5
*Cancer patients treated annually*	
Median (inter-quartile range: IQR)	70 (20–100)
*Cancer patients with pain treated annually*	
Median (IQR)	3 (0–10)
*Working facility*	
Designated cancer hospital/ University hospital	374 (67.5%)
Others	180 (32.5%)
*Primary practice area*	
Interventional radiology	194 (35.0%)
Diagnostic radiology	291 (52.5%)
Radiation oncology	10 (1.8%)

Current Implementation.

Table 2 summarizes the current implementation status and the number of procedures performed in the past three years for each treatment. The proportions of respondents currently performing nerve block, bone augmentation, and TAE were 18.1%, 22.6%, and 51.4%, respectively. The proportions performing ≥ 10 procedures over the past three years were 1.3%, 2.5%, and 3.2%, respectively.

**Table 2:** Implementation of palliative IR procedures (*N* = 554)

	*N* (%)
*Celiac plexus / splanchnic nerve block for pain due to pancreatic cancer*	
Currently implementing	100 (18.1%)
Not implementing	444 (80.1%)
No response	10 (1.8%)
*Number of procedures in the past three years*	
0	487 (87.9%)
1–9	43 (7.8%)
10 ≤	7 (1.3%)
No response	17 (3.1%)
*Percutaneous vertebroplasty/ osteoplasty for pain due to bone metastasis*	
Currently implementing	125 (22.6%)
Not implementing	423 (76.4%)
No response	6 (1.1%)
*Number of procedures in the past three years*	
0	466 (84.1%)
1–9	60 (10.8%)
10 ≤	14 (2.5%)
No response	14 (2.5%)
*Transcatheter arterial embolization for pain due to bone metastasis*	
Currently implementing	285 (51.4%)
Not implementing	262 (47.3%)
No response	7 (1.3%)
*Number of procedures in the past three years*	
0	398 (71.8%)
1–9	125 (22.6%)
10 ≤	18 (3.2%)
No response	13 (2.3%)

Factors Associated with Non-implementation (Barriers).

Tables 3, 4 and 5 show the results of the univariable and multivariable logistic regression analyses evaluating factors associated with non-implementation of each procedure. In this analysis, barriers were defined as factors independently associated with non-implementation of the procedure. Only factors that remained significant in the multivariable analyses are described below. Across all three procedures, lack of adequate training emerged as the most consistent and prominent barrier to implementation. This was strongly associated with non-implementation of nerve block (OR = 0.063 [95% CI, 0.026–0.153], *p* < 0.001), bone augmentation (OR = 0.058 [95% CI, 0.025–0.134], *p* < 0.001), and TAE (OR = 0.189 [95% CI, 0.097–0.367], *p* < 0.001). For nerve block, additional independent barriers included lack of timely patient referrals (OR = 3.301 [95% CI, 1.195–9.123], *p* = 0.021), lack of confidence in managing complications (OR = 0.180 [95% CI, 0.065–0.502], *p* = 0.001), limited case volume (OR = 0.215 [95% CI, 0.076–0.607], *p* = 0.004), lack of time for procedures (OR = 3.541 [95% CI, 1.354–9.260], *p* = 0.010), and the need for large-scale domestic evidence (OR = 2.992 [95% CI, 1.330–6.730], *p* = 0.008); age was also significantly associated (OR = 2.224 [95% CI, 1.045–4.733], *p* = 0.038). For bone augmentation, an additional independent barrier was lack of confidence in managing complications (OR = 0.232 [95% CI, 0.096–0.563], *p* = 0.001). For TAE, additional barriers included lack of timely patient referrals (OR = 0.143 [95% CI, 0.046–0.444], *p* < 0.001), lack of time for procedures (OR = 2.164 [95% CI, 1.183–3.961], *p* = 0.012), and lack of institutional resources (OR = 4.556 [95% CI, 1.672–12.416], *p* = 0.003); age (OR = 1.958 [95% CI, 1.215–3.157], *p* = 0.006) and annual number of cancer patients with pain (OR = 1.424 [95% CI, 1.105–1.834], *p* = 0.006) were also significantly associated.

**Table 3:** Factors associated with non-implementation of Celiac plexus/splanchnic nerve block

Variables		Number of responses	Univariable analysis				Multivariable analysis	
			Implementing	Not implementing	OR (95% CI)	*P* value	OR (95% CI)	*P* value
*Demographics*								
Gender	Female	42	5	37	Reference		1.659 (0.371–7.418)	0.508
	Male	501	94	407	1.709 (0.654–4.466)	0.274		
Age	− 39	104	15	89	Reference		2.224 (1.045–4.733)	0.038*
	40–59	364	64	300	0.790 (0.429–1.454)	0.449		
	60 -	74	20	54	0.455 (0.215–0.963)	0.040*		
Number of cancer patients with pain treated annually	0	192	16	176	Reference		1.319 (0.910–1.911)	0.143
	1–9	144	30	114	0.345 (0.180–0.662)	0.001*		
	10–49	122	29	94	0.292 (0.151–0.564)	< 0.001*		
	50 -	53	18	35	0.177 (0.082–0.380)	< 0.001*		
Working facility	Designated cancer hospital/ University hospital	366	71	295	0.809 (0.503-1.300)	0.381		
	Others	178	29	149	Reference			
Primary practice area	Interventional radiology	191	54	137	Reference		1.081 (0.567–2.061)	0.814
	Diagnostic radiology	285	34	251	2.910 (1.806–4.688)	< 0.001*		
	Others	36	4	32	3.153 (1.064–9.342)	0.038*		
*Barriers*								
Timely patient referrals	Neutral or disagree	473	77	396	Reference		3.301 (1.195–9.123)	0.021*
	Agree	77	23	25	4.731 (2.554–8.766)	< 0.001*		
Other treatments considered sufficient for pain relief	Neutral or disagree	462	86	376	Reference			
	Agree	65	13	52	1.093 (0.570–2.097)	0.789		
Adequate training received	Neutral or disagree	444	36	408	Reference		0.063 (0.026–0.153)	< 0.001*
	Agree	86	64	22	32.970 (18.235–59.611)	< 0.001*		
Confident in managing complications	Neutral or disagree	451	46	405	Reference		0.180 (0.065–0.502)	0.001*
	Agree	80	54	26	18.286 (10.461–31.964)	< 0.001*		
Limited case volume hampers skill development	Neutral or disagree	112	29	83	Reference		0.215 (0.076–0.607)	0.004*
	Agree	421	71	350	0.581 (0.354–0.951)	0.031*		
Lack of time for procedures	Neutral or disagree	279	79	200	Reference		3.541 (1.354–9.260)	0.010*
	Agree	251	21	230	0.231 (0.138–0.388)	< 0.001*		
Difficulty in interdepartmental coordination	Neutral or disagree	327	71	256	Reference		1.105 (0.473–2.583)	0.817
	Agree	203	29	174	0.601 (0.374–0.964)	0.035*		
No intra-departmental consensus	Neutral or disagree	416	91	325	Reference		3.770 (0.923–15.399)	0.065
	Agree	114	9	105	0.306 (0.149–0.628)	0.001*		
Lack of institutional approval	Neutral or disagree	440	91	349	Reference		0.398 (0.082–1.923)	0.251
	Agree	89	9	80	0.431 (0.209–0.892)	0.023*		
Lack of institutional resources	Neutral or disagree	368	93	275	Reference		3.265 (0.721–14.787)	0.125
	Agree	101	3	98	0.091 (0.028–0.292)	< 0.001*		
Need for large-scale domestic evidence	Neutral or disagree	194	49	145	Reference		2.992 (1.330–6.730)	0.008*
	Agree	337	51	286	0.528 (0.340–0.819)	0.004*		
Centralization preferred	Neutral or disagree	243	59	184	Reference		1.982 (0.884–4.441)	0.097
	Agree	291	41	250	0.511 (0.329–0.795)	0.003*		

**Table 4:** Factors associated with non-implementation of vertebroplasty/osteoplasty

Variables		Number of responses	Univariable analysis					Multivariable analysis	
			Implementing	Not implementing	OR (95% CI)	*P* value		OR (95% CI)	*P* value
*Demographics*									
Gender	Female	42	6	36	Reference			0.707 (0.146–3.429)	0.666
	Male	505	119	386	1.850 (0.761–4.497)	0.175			
Age	-39	107	17	90	Reference			1.039 (0.501–2.154)	0.919
	40–59	363	89	274	1.720 (0.972–3.043)	0.063			
	60 -	76	19	57	1.765 (0.847-3. 675)	0.129			
Number of cancer patients with pain treated annually	0	194	23	171	Reference			1.015 (0.677–1.522)	0.941
	1–9	145	36	109	2.456 (1.381–4.367)	0.002*			
	10–49	121	43	78	4.099 (2.311–7.268)	< 0.001*			
	50 -	55	18	37	3.617 (1.775–7.370)	< 0.001*			
Working facility	Designated cancer hospital/ University hospital	371	97	274	1.884 (1.183-3.000)	0.008*		1.228 (0.510–2.960)	0.647
	Others	177	28	149	Reference				
Primary practice area	Interventional radiology	190	70	120	Reference			0.526 (0.275–1.004)	0.052
	Diagnostic radiology	291	40	251	0.273 (0.175–0.426)	< 0.001*			
	Others	35	6	29	0.355 (0.140–0.896)	0.028*			
*Barriers*									
Timely patient referrals	Neutral or disagree	467	98	369	Reference			0.440 (0.168–1.154)	0.095
	Agree	67	27	40	2.542 (1.486–4.346)	< 0.001*			
Other treatments considered sufficient for pain relief	Neutral or disagree	455	105	350	Reference				
	Agree	78	19	59	1.073 (0.612–1.881)	0.804			
Adequate training received	Neutral or disagree	392	26	366	Reference			0.058 (0.025–0.134)	< 0.001*
	Agree	143	44	99	31.673 (18.582–53.985)	< 0.001*			
Confident in managing complications	Neutral or disagree	409	37	372	Reference			0.232 (0.096–0.563)	0.001*
	Agree	128	88	40	22.119 (13.365–36.608)	< 0.001*			
Limited case volume hampers skill development	Neutral or disagree	369	64	296	Reference			1.303 (0.588–2.887)	0.515
	Agree	176	61	115	0.408 (0.270–0.615)	< 0.001*			
Lack of time for procedures	Neutral or disagree	303	95	208	Reference			1.618 (0.711–3.678)	0.251
	Agree	232	29	203	0.313 (0.198–0.495)	< 0.001*			
Difficulty in interdepartmental coordination	Neutral or disagree	297	82	215	Reference			0.937 (0.427–2.056)	0.870
	Agree	239	43	196	0.575 (0.379–0.873)	0.009*			
No intra-departmental consensus	Neutral or disagree	421	111	310	Reference			1.733 (0.558–5.379)	0.342
	Agree	115	14	101	0.387 (0.213–0.705)	0.002*			
Lack of institutional approval	Neutral or disagree	439	113	326	Reference			2.515 (0.683–9.263)	0.166
	Agree	96	11	85	0.373 (0.192–0.725)	0.004*			
Lack of institutional resources	Neutral or disagree	327	92	235	Reference			0.724 (0.305–1.718)	0.463
	Agree	142	22	120	0.468 (0.280–0.783)	0.004*			
Need for large-scale domestic evidence	Neutral or disagree	207	67	140	Reference			1.823 (0.833–3.988)	0.133
	Agree	329	57	272	0.438 (0.291–0.658)	< 0.001*			
Centralization preferred	Neutral or disagree	226	60	166	Reference				
	Agree	312	65	247	0.728 (0.487–1.089)	0.122			

**Table 5:** Factors associated with non-implementation of percutaneous transcatheter arterial embolization (TAE)

Variables		Number of responses	Univariable analysis					Multivariable analysis	
			Implementing	Not implementing	OR (95% CI)	*P* value		OR (95% CI)	*P* value
*Demographics*									
Gender	Female	41	17	24	Reference			0.999 (0.396–2.521)	0.999
	Male	505	268	237	1.598 (0.837–3.044)	0.155			
Age	-39	106	39	67	Reference			1.958 (1.215–3.157)	0.006*
	40–59	364	193	171	0.516 (0.330–0.805)	0.004*			
	60 -	75	52	23	0.257 (0.137–0.483)	< 0.001*			
Number of cancer patients with pain treated annually	0	192	62	130	Reference			1.424 (1.105–1.834)	0.006*
	1–9	145	96	49	0.243 (0.154–0.385)	< 0.001*			
	10–49	122	72	50	0.331 (0.207–0.530)	< 0.001*			
	50 -	55	40	15	0.179 (0.092–0.348)	< 0.001*			
Working facility	Designated cancer hospital/ University hospital	368	200	168	0.760 (0.531–1.086)	0.132			
	Others	179	85	94	Reference				
Primary practice area	Interventional radiology	192	138	54	Reference			0.658 (0.425–1.021)	0.062
	Diagnostic radiology	287	115	172	3.822 (2.579–5.664)	< 0.001*			
	Others	36	17	19	2.856 (1.382–5.903)	0.005*			
*Barriers*									
Timely patient referrals	Neutral or disagree	481	234	247	Reference			0.143 (0.046–0.444)	< 0.001*
	Agree	54	50	4	13.194 (4.692–37.107)	< 0.001*			
Other treatments considered sufficient for pain relief	Neutral or disagree	422	230	192	Reference				
	Agree	114	54	60	0.751 (0.496–1.137)	0.176			
Adequate training received	Neutral or disagree	242	61	181	Reference			0.189 (0.097–0.367)	< 0.001*
	Agree	295	223	72	9.190 (6.201–13.620)	< 0.001*			
Confident in managing complications	Neutral or disagree	292	103	189	Reference			0.907 (0.477–1.721)	0.764
	Agree	244	181	63	5.272 (3.627–7.662)	< 0.001*			
Limited case volume hampers skill development	Neutral or disagree	305	192	113	Reference			1.033 (0.592–1.801)	0.909
	Agree	232	92	140	0.387 (0.272–0.549)	< 0.001*			
Lack of time for procedures	Neutral or disagree	381	230	151	Reference			2.164 (1.183–3.961)	0.012*
	Agree	155	54	101	0.351 (0.238–0.518)	< 0.001*			
Difficulty in interdepartmental coordination	Neutral or disagree	356	200	156	Reference			1.066 (0.595–1.909)	0.829
	Agree	181	85	96	0.691 (0.482–0.989)	0.043*			
No intra-departmental consensus	Neutral or disagree	438	243	195	Reference			0.646 (0.300–1.390)	0.264
	Agree	98	41	57	0.577 (0.370–0.899)	0.015*			
Lack of institutional approval	Neutral or disagree	468	263	205	Reference			1.604 (0.612–4.209)	0.337
	Agree	67	21	46	0.356 (0.206–0.615)	< 0.001*			
Lack of institutional resources	Neutral or disagree	423	241	182	Reference			4.556 (1.672–12.416)	0.003*
	Agree	53	11	42	0.198 (0.099–0.395)	< 0.001*			
Need for large-scale domestic evidence	Neutral or disagree	153	81	72	Reference				
	Agree	383	203	180	1.002 (0.689–1.459)	0.990			
Centralization preferred	Neutral or disagree	276	161	115	Reference			1.316 (0.787–2.199)	0.295
	Agree	260	123	137	0.641 (0.456–0.902)	0.011*			

Perceptions of Palliative IR Procedures.

Table 6 summarizes respondents’ willingness to perform each procedure. “Willing to continue” referred to respondents who were currently performing the procedure and intended to continue, whereas “willing to implement” referred to those not currently performing the procedure but expressing willingness to adopt it in the future. Overall willingness (willing to continue or implement) was reported by 50.2% for nerve block, 48.4% for bone augmentation, and 66.0% for TAE.

**Table 6:** Willingness to perform palliative IR procedures in the future (*N* = 554)

	No.
*Celiac plexus / splanchnic nerve block for pain due to pancreatic cancer*	
Continue performing	51 (9.2%)
Start performing	227 (41.0%)
Prefer not to perform	75 (13.5%)
Do not plan to perform	185 (33.4%)
No response	16 (2.9%)
*Percutaneous vertebroplasty/ osteoplasty for pain due to bone metastasis*	
Continue performing	69 (12.5%)
Start performing	199 (35.9%)
Prefer not to perform	119 (21.5%)
Do not plan to perform	157 (28.3%)
No response	10 (1.8%)
*Transcatheter arterial embolization for pain due to bone metastasis*	
Continue performing	131 (23.6%)
Start performing	235 (42.4%)
Prefer not to perform	81 (14.6%)
Do not plan to perform	99 (17.9%)
No response	8 (1.4%)

Perceived effectiveness was high for nerve block (81.2% agree) and bone augmentation (83.1% agree), but lower for TAE (62.1% agree), with the remainder reporting neutral or disagreeing responses.

## Discussion

This nationwide survey of board-certified interventional radiologists in Japan showed that implementation of palliative IR procedures for refractory cancer pain remains limited, particularly for nerve block (18.1%) and bone augmentation (22.6%), while TAE was more common (51.4%). Lack of adequate training emerged as a shared barrier, with additional procedure-specific physician- and system-level factors contributing to non-implementation, particularly for nerve block and TAE. Although most respondents recognized their effectiveness, especially nerve block and bone augmentation, willingness to perform was lower, revealing a gap between perceived benefit and practice.

In Western countries, palliative IR procedures for cancer pain are also underutilized. In the United States, surveys of pain physicians show that few patients with refractory cancer pain receive neurolytic blocks or intrathecal analgesia, mainly due to low referral rates despite broad recognition of their value [15]. Bone augmentation is widely practiced, but mostly for osteoporotic fractures rather than metastatic pain [16]. In the United Kingdom, a national survey estimated that about 12,000 cancer patients could benefit from advanced pain interventions, yet fewer than 1,000 were performed annually, with many centers lacking access [17]. To address these gaps, European societies have called for routine access to multidisciplinary pain teams [18]. More recent international reviews emphasize that palliative and musculoskeletal interventional oncology techniques are rapidly expanding, supported by accumulating evidence and guideline integration; however, they also highlight persistent challenges in implementation, including the need for specialized training, multidisciplinary coordination, and institutional support [19, 20]. Importantly, low referral is not unique to Western countries. Previous studies from Japan have also reported limited referrals and restricted availability of palliative interventional procedures, even when potentially eligible patients were present [11, 12]. Our study further demonstrates similarly low implementation rates by IR physicians in Japan, particularly for nerve block and bone augmentation, with lack of adequate training and institutional barriers contributing. Low referral rates and training barriers should not be viewed as independent issues but as closely interrelated factors. Limited training opportunities among interventional radiologists may reduce procedural availability and confidence, which in turn discourages referrals from palliative care teams and other clinicians. Conversely, persistently low referral volumes further limit opportunities for skill acquisition and experience. Thus, our findings suggest that training-related barriers may represent a shared underlying mechanism contributing to low referral rates across different healthcare systems, including both Japan and Western countries.

Despite all respondents being board-certified interventional radiologists, 52.5% reported diagnostic imaging as their main practice, and only 35.0% were dedicated to IR. A recent Japanese survey similarly found that 56.6% of interventional radiologists devoted less than 25% of their work time to IR [21]. This likely reflects the Japanese model in which many IR physicians also perform diagnostic reading, often prioritized for reimbursement. This overlap may explain why “lack of time for procedures” emerged as a significant barrier for TAE and bone augmentation. However, this interpretation should be considered hypothesis-generating, as the present study did not assess changes in overall IR procedure volumes.

Insufficient training opportunities were a common barrier. Although these procedures are not technically complex compared with other advanced IR interventions, their safe implementation requires disease-specific knowledge, appropriate patient selection, and familiarity with complication management, which may be challenging for operators without regular clinical exposure. Even for TAE, adopted by about half of respondents, lack of adequate training remained a major barrier, likely because TAE is usually performed for hepatocellular carcinoma or bleeding rather than pain palliation.

The reasons why institutional factors were perceived as barriers remain unclear and warrant further study. Univariable analysis also suggested that negotiation with other departments was a concern, likely reflecting limited evidence and guideline coverage, which may reduce referrals, especially when no in-house experts are available [12].

A clear gap emerged between recognition of effectiveness and willingness to perform. Although over 80% of respondents endorsed the value of nerve block and bone augmentation, only about half were willing to perform them. This likely reflects limited experience, concern about complications, and lack of reimbursement incentives. Bridging this gap will require multifaceted strategies: society-led training and tele-mentoring to expand expertise [22], multidisciplinary collaboration for case selection, and improved access to information on available centers (e.g., JSIR “Hospital Search System” [23], SIR “Doctor Finder” [24]). Public awareness through patient-oriented resources, such as CIRSE’s website [25] and JSIR educational videos [26], will also be essential for broader dissemination of palliative IR procedures. Such approaches are realistic, as recent international reviews describe successful integration of palliative IR procedures into multidisciplinary oncology care pathways supported by professional societies [19, 20].

This study has limitations. The response rate was moderate (51.1%), raising the possibility of nonresponse bias. Respondents may have been more interested in or aware of palliative IR procedures than nonrespondents, potentially leading to overestimation of implementation and willingness. Self-reported data may be affected by recall or reporting bias, and the characteristics of nonrespondents are unknown. In addition, the survey focused on selected palliative IR procedures and did not include other ablative or neurolytic techniques, which limits the generalizability of the results. The survey (February–March 2020) was conducted during the early phase of the COVID-19 pandemic in Japan, which may have influenced responses regarding time constraints and institutional resources. In addition, because the survey was conducted in 2020, the findings may not fully reflect subsequent changes in palliative IR practice, including potential improvements in awareness, training opportunities, or institutional support. The present results should therefore be interpreted as a baseline assessment of nationwide practice patterns at that time. Moreover, the survey did not assess clinical outcomes or patient-level data; future research should include prospective studies.

In conclusion, the implementation of palliative IR procedures in Japan remains limited, despite the high perceived effectiveness among IR physicians. Lack of adequate procedural training was the most consistent barrier across all procedures, suggesting that greater availability of training opportunities may help narrow the gap between perceived benefit and actual practice. These findings highlight a clinically important mismatch: procedures viewed as effective for refractory cancer pain are not reaching patients who might benefit from them.

## Data Availability

The datasets generated and/or analyzed during the current study are not publicly available due to ethical and privacy considerations, but are available from the corresponding author on reasonable request.

## References

[1] Evenepoel M, Haenen V, De Baerdemaecker T, et al. Pain prevalence during cancer treatment: A systematic review and Meta-Analysis. J Pain Symptom Manage. 2022;63(3):e317–35. 34563628 10.1016/j.jpainsymman.2021.09.011

[2] van den Beuken-van Everdingen MH, de Rijke JM, Kessels AG, Schouten HC, van Kleef M, Patijn J. Prevalence of pain in patients with cancer: a systematic review of the past 40 years. Ann Oncol. 2007;18(9):1437–49. 10.1093/annonc/mdm056 17355955

[3] van den Beuken-van Everdingen MH, Hochstenbach LM, Joosten EA, Tjan-Heijnen VC, Janssen DJ. Update on prevalence of pain in patients with cancer: systematic review and Meta-Analysis. J Pain Symptom Manage. 2016;51(6):1070–90. e1079. 10.1016/j.jpainsymman.2015.12.340 27112310

[4] Loveday BA, Sindt J. Ketamine protocol for palliative care in cancer patients with refractory pain. J Adv Pract Oncol. 2015;6(6):555–61. 27648345 PMC5017546

[5] Afsharimani B, Kindl K, Good P, Hardy J. Pharmacological options for the management of refractory cancer pain-what is the evidence? Support Care Cancer. 2015;23(5):1473–81. 25749509 10.1007/s00520-015-2678-9

[6] Birthi P, Sloan P. Interventional treatment of refractory cancer pain. Cancer J. 2013;19(5):390–6. 24051611 10.1097/PPO.0b013e3182a631a2

[7] Christo PJ, Mazloomdoost D. Interventional pain treatments for cancer pain. Ann N Y Acad Sci. 2008;1138:299–328. 18837908 10.1196/annals.1414.034

[8] Tsao MN, Barnes EA, Chow E. Multiple bone metastases: what the palliative care specialist should know about the potential, limitations and practical aspects of radiation therapy. Ann Palliat Med. 2020;9(3):1307–13. 31431026 10.21037/apm.2019.07.09

[9] Filippiadis DK, Cornelis FH, Kelekis A. Interventional oncologic procedures for pain palliation. Presse Med. 2019;48(7–8 Pt 2):e251–6. 31447338 10.1016/j.lpm.2019.06.006

[10] Swarm RA, Youngwerth JM, Agne JL et al. Adult cancer Pain, version 2.2025, NCCN clinical practice guidelines in oncology. J Natl Compr Canc Netw, 2025;23(7). 10.6004/jnccn.2025.0032 40639401

[11] Uehara Y, Matsumoto Y, Kosugi T, et al. Availability of and factors related to interventional procedures for refractory pain in patients with cancer: a nationwide survey. BMC Palliat Care. 2022;21(1):166. 36154936 10.1186/s12904-022-01056-6 PMC9511722

[12] Matsumoto Y, Uehara Y, Mizushima A, et al. Availability of, barriers to Performing, and educational practices of interventional procedures for refractory pain in cancer patients: A nationwide survey of designated cancer hospitals in Japan. Palliat Med Rep. 2024;5(1):543–52. 39758849 10.1089/pmr.2024.0028 PMC11693962

[13] Filippiadis D, Tutton S, Kelekis A. Pain management: the rising role of interventional oncology. Diagn Interv Imaging. 2017;98(9):627–34. 28739433 10.1016/j.diii.2017.06.015

[14] Elhakim M, Dexter F, Pearson ACS. US critical access hospitals’ listings of pain medicine physicians and other clinicians performing interventional pain procedures. J Clin Anesth. 2019;58:52–4. 31077934 10.1016/j.jclinane.2019.05.005

[15] Partain DK, Santivasi WL, Kamdar MM, et al. Attitudes and beliefs regarding pain medicine: results of a National palliative physician survey. J Pain Symptom Manage. 2024;68(2):115–22. 38677489 10.1016/j.jpainsymman.2024.04.015

[16] Laratta JL, Shillingford JN, Lombardi JM, et al. Utilization of vertebroplasty and kyphoplasty procedures throughout the united States over a recent decade: an analysis of the nationwide inpatient sample. J Spine Surg. 2017;3(3):364–70. 29057344 10.21037/jss.2017.08.02 PMC5637187

[17] Kay S, Husbands E, Antrobus JH, Munday D. Provision for advanced pain management techniques in adult palliative care: a National survey of anaesthetic pain specialists. Palliat Med. 2007;21(4):279–84. 17656403 10.1177/0269216307078306

[18] Bennett MI, Eisenberg E, Ahmedzai SH, et al. Standards for the management of cancer-related pain across Europe-A position paper from the EFIC task force on cancer pain. Eur J Pain. 2019;23(4):660–8. 30480345 10.1002/ejp.1346 PMC7027571

[19] Key BM, Callstrom MR, Filippiadis D. Musculoskeletal interventional oncology: A contemporary review. AJR Am J Roentgenol. 2023;221(4):503–16. 37222277 10.2214/AJR.23.29110

[20] Tomasian A, Filippiadis DK, Tutton S, et al. Comprehensive palliative musculoskeletal interventional radiology care for patients with cancer. Radiographics. 2022;42(6):1654–69. 36190860 10.1148/rg.220009

[21] Sone M, Yasunaga H, Osawa M, et al. Impact of work environment on job satisfaction among interventional radiologists in japan: A Cross-sectional study. Interv Radiol (Higashimatsuyama). 2024;9(1):13–9. 38524998 10.22575/interventionalradiology.2023-0022 PMC10955479

[22] Oshima T, Sone M, Nishiofuku H, Arai Y, Satomi E. Feasibility of an interventional radiology telementoring support system using automated patient privacy artificial intelligence software. J Vasc Interv Radiol; 2025. 10.1016/j.jvir.2025.07.012 40706749

[23] Japanese Society of Interventional Radiology. Hospital Search System. https://ivr-search.jsir.or.jp/. Accessed December 20 2025.

[24] Society of Interventional Radiology. Doctor Finder. https://www.sirweb.org/doctor-finder/. Accessed December 20 2025.

[25] Cardiovascular and Interventional Radiological Society of Europe. https://www.cirse.org/patients/general-information/. Accessed December 20 2025.

[26] Japanese Society of Interventional Radiology. Educational Video for Patients. https://www.jsir.or.jp/kaiin/movie/. Accessed December 20 2025.

